# The Role of Microbiomes in Pregnant Women and Offspring: Research Progress of Recent Years

**DOI:** 10.3389/fphar.2020.00643

**Published:** 2020-05-08

**Authors:** Yao Yao, Xiaoyu Cai, Chunyan Chen, Hui Fang, Yunchun Zhao, Weidong Fei, Fengying Chen, Caihong Zheng

**Affiliations:** ^1^Department of Pharmacy, Women's Hospital, School of Medicine, Zhejiang University, Hangzhou, China; ^2^Department of Pharmacy, Hangzhou First People's Hospital, Hangzhou, China; ^3^Department of Pharmacy, Sir Run Run Shaw Hospital, School of Medicine, Zhejiang University, Hangzhou, China; ^4^Department of Pharmacy, The Affiliated Hospital of Hangzhou Normal University, Hangzhou, China

**Keywords:** pregnancy, complication, microbiomes, offspring, mechanism

## Abstract

Pregnancy is a complicated and delicate process, the maternal body undergoes changes on hormones, immunity, and metabolism during pregnancy to support fetal development. Microbiomes in the human body mainly live in the intestine, and the human gut microbiomes are complex, which composed of more than 500 to 1500 different bacteria, archaea, fungi, and viruses. Studies have shown that these microbiomes are not only involved in the digestion and absorption of food but also indispensable in regulating host health. In recent years, there has been increasing evidence that microbiomes are important for pregnant women and fetuses. During pregnancy, there will be great changes in gut microbiomes. Regulating gut microbiomes is beneficial to the health of the mother and the fetus. In addition, many complications during pregnancy are related to gut microbiomes, such as gestational diabetes, obesity, preeclampsia, digestive disorders, and autoimmune diseases. Moreover, the microbiomes in mother's milk and vagina are closely related to the colonization of microbiomes in the early life of infants. In this review, we systematically review the role of maternal microbiomes in different gestational complications, and elucidate the function and mechanism of maternal microbiomes in the neural development and immune system of offspring. These will provide a clear knowledge framework or potential research direction for researchers in related fields.

## Introduction

Pregnancy is a unique event with a lot of hormonal, immune, and metabolic changes in the body (Zhang et al., 2019). Some changes of the body during pregnancy may be closely related to the changes in the composition and diversity of gut microbiomes (GMs) (Barbour et al., 2007; Gohir et al., 2015; Khan et al., 2019). Changes in the composition and diversity of GMs may ultimately affect the host's immunity, metabolism, digestion, and neurodevelopment (Carding et al., 2015; Pedersen et al., 2016). Therefore, focusing on the GMs may yield a positive insight into pregnancy. The composition and diversity of the maternal GMs change progressively every 3 months of the gestational period (Smid et al., 2018). However, GMs are basically unchanged during the first 3 months of pregnancy, such as Gram-positive and Gram-negative bacteria (de Brito Alves et al., 2019). The human gastrointestinal tract contains approximately 500~1,000 microbiomes and more than 3 million genotypes (Lozupone et al., 2012). The ratio of microbial cells to human cells is 10:1, and the genotype ratio is 100:1 (Vrieze et al., 2010). Researches showed that GMs play a vital role in human immunity and digestion, and are involved in the development of chronic diseases such as diabetes, hypertension, and inflammatory bowel disease (Hooper et al., 2012; Dolan and Chang, 2017; Lezutekong et al., 2018; Zhao et al., 2018). Increasing evidence indicated that the structure and composition of GMs are generally determined by the host's genetic factors, environmental factors, and dietary habits (David et al., 2014; Rothschild et al., 2018). During pregnancy, changes in the diversity and composition of GMs may occur naturally in multiple parts of the mother's body, including the oral cavity, vagina, intestine, and breast milk (Nuriel-Ohayon et al., 2016; Zhou and Xiao, 2018). However, the relationship between maternal GMs and the complex physiological conditions during pregnancy is still insufficiently discussed. Many scholars have begun to study the relationship between GMs imbalance and complications during pregnancy.

Gestational GMs are not only closely related to the health of pregnant women but also affect the health of offspring. The most important link between mother and fetus is the placenta, and placental microbiomes include *Tenericutes*, *Fusobacteria*, *Bacteroidetes*, and *Firmicutes*, all of which are nonpathogenic bacteria (Aagaard et al., 2014). However, factors such as maternal gestational diabetes, obesity, vaginal infections, and the use of antibiotics have led to changes in the composition of the placental microbiomes (Pelzer et al., 2017). For example, recent studies have reported that excessive weight gain during pregnancy is associated with risk factors such as disorders of placental microbiomes and preterm birth (Antony et al., 2015). In addition, in the postpartum environment, the relationship between the GMs of infant and breast milk microbiomes has also attracted increasing attention. A prospective study showed that the bacterial species in mother's breast milk are the most abundant in the GMs of offspring in the first month after the baby was born (Pannaraj et al., 2017), suggesting that breastfeeding is closely related to GMs of the infant in early life.

Therefore, it can be reasonably inferred that the strategy of regulating maternal GMs during the prenatal and early postnatal life of infants can be used as an innovative treatment method to prevent and/or control adverse maternal complications and offspring health. In this way, the use of probiotic interventions to regulate maternal microbiomes has become a safe strategy that can restore the community of symbiotic microbiomes and provide beneficial effects on maternal, fetal, and infant health (Sanz, 2011; Swartwout and Luo, 2018).

In this review, we introduce the role of GMs in different gestational complications and the function of maternal microbiomes in offspring. The factors that influence the microbiomes of pregnant women may provide a novel therapeutic target in the future. Understanding the adaptations of microbiomes and the mechanisms of how they can be modulated may be beneficial in gestational management.

## The Role of GMs in Gestational Complications

### Gestational Diabetes Mellitus

There are two types of diabetes during pregnancy, however, only Gestational diabetes mellitus (GDM) is discussed within this section so we would simply omit the other type. GDM is diabetes with normal glucose metabolism or impaired glucose tolerance before pregnancy, which occurs or is diagnosed only during pregnancy and is associated with many adverse maternal and neonatal outcomes (Group et al., 2008; Schneider et al., 2011; American Diabetes, 2018). More than 80% of diabetic pregnant women have GDM, the incidence of GDM in countries around the world is reported as 1% to 14%, and the incidence rate in China is 1% to 5%, which has increased significantly in recent years (Sacks et al., 2012; Zhu and Zhang, 2016). GDM patients with glucose metabolism can return to normal after delivery, but they will have an increased chance of developing type 2 diabetes in the future (Lauenborg et al., 2004; Bellamy et al., 2009). The clinical course of diabetic pregnant woman is complex, both mother and child have risk, should be given attention (Vohr and Boney, 2008; Damm et al., 2016).

In recent years, the role of GMs in GDM has received increasing attention. Reports showed that, compared with pregnant women with normal blood sugar, the abundance of *Parabacteroides*, *Dialister*, *Akkermansia*, *Roseburia*, *Bacteroides*, *Methanobrevibacter smithii*, *Eubacterium species*, *Alistipes species*, *Bifidobacterium species* was reduced (Kuang et al., 2017; Cortez et al., 2019), and the abundance of *Firmicutes*, *Klebsiella variicola*, *Collinsella*, *Rothia*, *Ruminococcus*, *Actinobacteria*, *Parabacteroides distasonis*, *Desulfovibrio* was increased (Crusell et al., 2018; Ferrocino et al., 2018). Moreover, one study showed that placental microbiomes in patients with GDM changed significantly compared to those with normal blood sugar (Bassols et al., 2016). Compared with healthy pregnant women, the relative abundance of *Pseudomonadales order* and *Acinetobacter genus* in the intestinal flora of GDM patients is reduced. The GMs of patients diagnosed with GDM in the third trimester have previously exhibited a typical flora disorder, which persists even after about 8 months postpartum (Crusell et al., 2018). These data suggest that GMs may play an important role in GDM. Adverse pregnancy complications in pregnant women are closely related to intestinal permeability, such as GDM, insulin resistance, inflammatory response and hyperglycemia (Flint et al., 2012; Navab-Moghadam et al., 2017). In general, the imbalance of GMs is the main cause of impaired intestinal permeability (Navab-Moghadam et al., 2017). In addition, the study by Bassols et al. showed that the reduced relative abundance of *Acinetobacter genus* is related to a decrease in the blood eosinophil count and a decrease in the expression of several antiinflammatory cytokines in the placenta, such as metallopeptidase inhibitor 3 and IL-10 (Bassols et al., 2016). Therefore, GMs may predict the development and prognosis of GDM. Properly regulating the GMs of pregnant women may be an effective method for the treatment of GDM. However, relevant mechanistic or interventional trials are still lacking and further prospective trials are needed.

### Gestational Obesity

Gestational obesity generally refers to obesity caused by a woman's weight gain exceeding the normal weight gain range during pregnancy. Gestational obesity can be divided into two phases: obesity during pregnancy and postpartum obesity (postpartum weight retention). The standards adopted by the American Institute of Medical Research (IOM) in 2009 are used in China to determine whether the degree of weight gain during pregnancy is abnormal (**Table 1**). Gestational obesity not only adversely affects pregnant women, but also the fetus, including GDM, hypertension during pregnancy, subinvolution of uterus, giant baby, and neonatal congenital defects (Poston et al., 2016; Zambrano et al., 2016).

**Table 1:** Criteria for obesity during pregnancy in China.

Weight status before pregnancy	Weight gain during pregnancy		
	Insufficient weight gain during pregnancy (kg)	Normal gain during pregnancy (kg)	Overweight gain during pregnancy (Gestational obesity) (kg)
Thin	<12.5	12.5~18.0	>18.0
Normal	<11.5	11.5~16.0	>16.0
Overweight	<7.0	7.0~11.5	>11.5
Fat	<5.0	5.0~9.0	>9.0

Over the past few decades, it has been generally agreed that the main factor associated with gestational obesity is hormones. However, in recent years, the relationship between GMs and gestational obesity has received widespread attention. GMs during pregnancy can be regarded as a metabolic organ of the human body, and its imbalance may cause obesity and related metabolic disorders (Kalliomaki et al., 2008). A prospective follow-up study showed that excessive weight gain during pregnancy was associated with a high concentration of *Bacteroides* spp. in the intestine (Collado et al., 2008). Santacruz et al. found that in the gut of overweight pregnant women, the abundance of *Enterobacteriaceae* and *Escherichia coli* decreased significantly, while the abundance of *Bacteroides* and *Bifidobacterium* increased (Santacruz et al., 2010). The study further showed that the abundance of these microbiomes is related to weight gain and biochemical parameters such as transferrin, high-density lipoprotein cholesterol, triglycerides, plasma cholesterol, and folic acid. These studies indicate that GMs are closely related to gestational obesity, and the intestine may be an important organ for regulating maternal lipid metabolism. Therefore, regulating GMs to improve maternal obesity may be a promising and novel strategy, and further prospective trials are needed.

However, the mechanism by which GMs improve gestational obesity is not clear. By consulting the literature, we have compiled the possible mechanisms that currently exist as follows (Collado et al., 2008; Kumar et al., 2014; Barlow et al., 2015; Khan et al., 2016; Soderborg et al., 2016; Gu et al., 2017): (1) Chronic inflammation results in endotoxin released into the blood after bacterial death leading to endotoxemia. For example, lipopolysaccharides (cell wall components of Gram-negative bacteria) are closely related to insulin resistance and inflammatory responses. (2) Disorder of GMs alters epigenetic modifications. (3) Decreased probiotics and increased harmful bacteria may increase the intestinal capacity for absorption and monosaccharide uptake. (4) Changes in gut microbial metabolites. For example, bacterial metabolites SCFAs can regulate hormone metabolism, inhibit the body's inflammatory response, and change the composition of intestinal immune cells. (5) Certain GMs can inhibit the production of adipose cytokines induced by fasting, which can inhibit protein lipase, leading to the accumulation of fat in peripheral tissues to form obesity. (6) Activation of liver endocannabinoid (eCB) and ChREBP/SREBP-1system. (7) Metabolism of primary bile acids in the small intestine to secondary bile acids.

In summary, GMs play a crucial role in gestational obesity, but its mechanism remains to be further explored. Taking advantage of the double-edged sword of GMs may yield unexpected results in managing gestational obesity.

### Preeclampsia

Preeclampsia refers to increased blood pressure and proteinuria after 20 weeks of pregnancy, and symptoms such as headache, dizziness, nausea, vomiting, and epigastric discomfort may also occur (Brown et al., 2018). It is the second leading cause of maternal death in the world currently (Huppertz, 2008; Ghulmiyyah and Sibai, 2012; Mol et al., 2016). The incidence of preeclampsia is about 5% of pregnant women, and it is more common in primiparas and pregnant women with hypertension and vascular disease (Hutcheon et al., 2011; Ananth et al., 2013). The cause of preeclampsia is not yet clear. At present, the related risk factors are weight, pregnancy hypertension, first pregnancy, age, and history of preeclampsia. However, the combination of these risk factors can only predict the occurrence of preeclampsia in clinical practice in 30% of pregnant women (Odibo et al., 2015; Mol et al., 2016).

In recent years, great breakthroughs have been made in the etiology of preeclampsia with the efforts of researchers. The study by Kell et al. has shown that the mechanisms leading to the development of preeclampsia include abnormal trophoblast invasion into the placenta, oxidative stress, antiangiogenic response, and increased proinflammatory cytokines (Kell and Kenny, 2016). In addition, researchers have found that the placenta plays a key role in the pathogenesis of preeclampsia and adversely affects the fetus. This may be due to obstructed utero-placental blood flow, which increases the risk of preterm and low birth weight fetuses (Salmani et al., 2014; Ilekis et al., 2016; Carter et al., 2017).

In addition to the above, GMs have recently been linked to the etiology of preeclampsia. The identification of placental microbiomes in pregnant women with preeclampsia indicates the abundance of several types of bacteria in the placenta is related to gastrointestinal infection, respiratory infection or periodontitis, respectively. For example, *Escherichia*, *Bacillus*, *Salmonella*, and *Listeria* are associated with gastrointestinal infections, *Anoxybacillus*, and *Klebsiella* are associated with respiratory infections, *Dialiste*, *Variovorax*, *Porphyromonas*, and *Prevotella shahii* are associated with periodontitis (Amarasekara et al., 2015). In addition, studies have shown that the abundance of pathogenic bacteria (*Bulleidia moorei* and *Clostridium perfringens*) in the intestine of preeclampsia women is increased, while the abundance of probiotics (*Coprococcus catus*) is decreased compared with healthy pregnant women (Lv et al., 2019). The above evidences suggest that: (1) The imbalance of placental microbiomes may be closely related to the occurrence and development of preeclampsia. (2) GMs play a vital role in the preeclampsia of pregnant women. Therefore, detecting intestinal and placental microbiomes in pregnant women with preeclampsia may provide a new idea for improving clinical symptoms. In addition, probiotic interventions may be an effective way to protect pregnant women from suffering headaches, dizziness, nausea, or vomiting. However, the above data only show that GMs is related to preeclampsia, and the specific mechanism remains to be further explored.

### Disease of Digestive Tract

The most common digestive tract diseases are tumors, as well as enteritis and gastritis. But the scope of our discussion here does not include tumors. Studies have reported that digestive tract management during pregnancy is the most challenging for gastroenterologists, at which time multiple reactions occur in the gastrointestinal tract, such as esophageal reflux, constipation, nausea, and vomiting (McCarthy et al., 2014). The incidence of vomiting during pregnancy is about 50%, and the incidence of nausea is about 50% to 80% (Matthews et al., 2015). A few pregnant women have severe early pregnancy reactions, frequent nausea, and vomiting, resulting in fluid imbalance and metabolic disorders, and even endanger the life of pregnant women, which is called hyperemesis gravidarum and the incidence is about 1.2% (Einarson et al., 2013). At present, the treatment of digestive tract diseases during pregnancy is mostly symptomatic, and the mechanism of their occurrence is unclear.

Studies on the relationship between digestive tract diseases and GMs have been available in the past, but few have studied the relationship between digestive tract diseases during pregnancy and GMs. Recent studies have shown that the relative abundance of *Blautia* and *Collinsella* in the intestine of pregnant women with gastrointestinal disorders is significantly reduced, and the abundance of *Acinetobacter*, *Enterococci*, and *Paenibacillus* is increased. The author further revealed that *Blautia* and *Collinsella* may be served as a new biomarker for digestive disorders in pregnancy (Jin et al., 2019). In addition, increasing evidences indicate that disorders of the maternal GMs increase the prevalence of colitis in the offspring in adulthood. Xie et al. found that high-fat diets in mice during pregnancy altered the intestinal flora of offspring and exacerbated dextran sodium sulfate (DSS)–induced colitis in offspring (Xie et al., 2018). Adult mice colonized with *Lactobacillus rhamnosus* (LGG) showed increased IgA production and decreased susceptibility to intestinal injury and inflammation induced in the dextran sodium sulphate model of colitis. Thus, neonatal colonization of mice with LGG enhances intestinal functional maturation and IgA production, and confers lifelong health consequences on protection from intestinal injury and inflammation (Yan et al., 2017). Not only that, a population-based cohort study suggested that exposure to antibiotics during pregnancy increases the risk of offspring from inflammatory bowel disease (Ortqvist et al., 2019).

To sum up, the GMs are closely related to maternal and offspring digestive tract diseases. However, the role of GMs in maternal digestive tract diseases has not yet been clearly elucidated. The effect of maternal GMs on offspring's digestive tract disease is well established. Improving the GMs imbalance during pregnancy and strengthening the management and monitoring of GMs may be a potential method for treating digestive diseases in pregnancy, which will reduce the probability of offspring suffering from digestive diseases.

### Autoimmune Diseases

Autoimmune diseases refer to the disease caused by the body's immune response to autoantigen, which leads to auto tissue damage such as rheumatoid arthritis (RA) and systemic lupus erythematosus (SLE) (Davidson and Diamond, 2001; Yao et al., 2018; Yao et al., 2019). There are more than 70 diseases classified as autoimmune diseases at present, and their prevalence in the general population is about 7% (Cooper et al., 2009). Many autoimmune diseases have a significantly higher incidence in women than men, including SLE and RA, which may be caused by female sex hormones regulating the immune system through sex hormone receptors (Adams Waldorf and Nelson, 2008; Cai et al., 2019; Tsao et al., 2019). During pregnancy, the mother undergoes a series of physiological changes to ensure the healthy growth of the fetus, including immune, hormonal, and metabolic changes (Kumar and Magon, 2012). In recent years, more and more evidences showed that there is a correlation between pregnancy and autoimmune diseases (Tincani et al., 2016).

Among the many factors related to pregnancy affecting autoimmune diseases, GMs have attracted widespread attention. It has been reported that the imbalance of GMs is associated with a variety of autoimmune diseases, such as SLE and RA (Mu et al., 2015). On the one hand, GMs disorder does exist in animal models (adjuvant arthritis model, collagen-induced arthritis model, lupus-prone mouse model) and human patients (Hevia et al., 2014; Zhang et al., 2014; Luo et al., 2018). On the other hand, the use of probiotics and antibiotics has been shown to regulate GMs (Mu et al., 2017a; Mu et al., 2017b; Manfredo Vieira et al., 2018). In a study of SLE, the authors found that changes in the GMs during pregnancy and lactation interfered with the autoimmune response (Mu et al., 2019). However, the study did not explore the mechanism of changes in GMs during pregnancy in SLE. Therefore, further prospective or interventional studies are needed to better understand the complex relationship between pregnancy and lupus. According to these reports, we speculate that intestinal microbiomes are closely related to RA in pregnancy, even though there are no published reports. A study on RA revealed that a single bacterium restores microbiomes imbalances to protect the bones of RA rats from damage (Pan et al., 2019). In addition, changes in the composition and structure of the GMs during pregnancy have been well recognized. Therefore, it is of far-reaching clinical significance to explore the role and mechanism of GMs in pregnancy with autoimmune diseases. GMs intervention for pregnant women with autoimmune disease may be a new clinical treatment, and further prospective trials are needed.

## The Role of Maternal Microbiomes in Offspring

### Effects of Maternal Microbiomes on the Offspring's Immune System

Maternal microbiomes are not only closely related to gestational complications, they are also vital to the health of the fetus. The development of babies' GMs is related to the existing microbiomes in many parts of mother's body. The microbiomes in these parts of the mother's body can be transmitted vertically to offspring, including the intestine, skin, breast milk, and vagina (Nyangahu et al., 2018). Studies have shown that the most significant periods are childbirth and postpartum, especially when infants are exposed to maternal skin, vagina, and feces (Mackie et al., 1999). In previous research, the idea that the uterine environment was sterile (Funkhouser and Bordenstein, 2013) has been questioned in recent years (Jimenez et al., 2008). Theis et al. found there are unique microbiomes in the placenta, which have not been reported before (Theis et al., 2019). However, recent evidence suggests that the microbiomes of pregnant women have a strong effect on the progeny microbiomes, whether or not placental microbiomes are actually present. Studies have expounded that the mother's GMs can better adapt to the baby's gut environment and live longer compared to nonmaternally derived flora (Ferretti et al., 2018). A mouse experiment also showed most of the bacteria in the meconium sample matched the mother's oral bacterial sample (genetically labeled) (Jimenez et al., 2008). These data reveal the importance of maternal microbiomes (not just GMs) to the fetus.

Furthermore, studies have shown that transient changes in maternal microbiomes during pregnancy drive the fetus's immune planning (Gomez de Aguero et al., 2016). A perinatal study showed that after treating pregnant mice with a large number of antibiotics, their offspring lacked IL-17–producing cells and IL-17 transcripts in the small intestine (Deshmukh et al., 2014). Similarly, in another study, after treating pregnant mice with three antibiotics, the offspring's GMs diversity reduced, and IL-17-IFN-γ–producing CD4^+^ and CD8^+^ cells in mesenteric lymph nodes decreased (Hu et al., 2015). These indicate that maternal microbiomes play an important role in enteric immune system of offspring.

In addition to the effects on the baby's enteric immune system, maternal microbiomes also affect the offspring's peripheral immune system. Gonzalez-Perez et al. observed that after treatment of dams with antibiotics during pregnancy and lactation, their pups lacked production of IFN-γ by CD8^+^ T cells, and the distribution of dendritic cells and NK cell subsets changed (Gonzalez-Perez et al., 2016). In a mouse model of autoimmunity based on the NLRP3 inflammasome mutation R258W, the maternal microbiomes were required for neonatal IL-1β and tumor necrosis factor-a (TNF-α) responses in the skin (Nakamura et al., 2012). The effect of perinatal maternal microbiomes on the immune system of offspring may be due to the effect of microbiomes on the development of immune cells (Josefsdottir et al., 2017). Joana Torres et al. found that aberrant microbiomes composition persists during pregnancy with IBD and alters the bacterial diversity and abundance in the infant stool (Torres et al., 2020). The research of Nyangahu et al. indicates that perturbations to maternal microbiomes dictate neonatal adaptive immunity (Nyangahu et al., 2018).

Therefore, from the data above, it can be seen that maternal microbiomes play a vital role in the offspring's immune system. Regulating the microbiomes of pregnant women is of great significance to the offspring's health, which may provide a novel insight into the management of pregnant women in clinical practice.

### Effects of Maternal Microbiomes on the Offspring's Neurodevelopment

The relationship between the gastrointestinal environment and the state of brain has been proven in the past, but the existence of the microbial-gut-brain axis has only received attention in the last decade (Bienenstock et al., 2015). There are many microbiomes are symbiotic with humans, these symbiotic microbiomes and their microbial groups are closely related to the host's brain and nerve development (Dinan and Cryan, 2017). In recent years, scholars found that the structural changes of microbiomes in the host are related to their neurological disorders, such as anxiety, autism, depression, and stress (Vuong and Hsiao, 2017). As early as 30 years ago, the role of the perinatal environment in heredity attracted attention, which could affect the offspring's health in adulthood (Barker, 2004). By understanding the Barker hypothesis, it has recently been further suggested that perinatal microbiomes play an important role in planning adult brain health (Codagnone et al., 2019).

Not only that, increasing evidence reveals microbiomes are critical in host neurodevelopment. A study of germ-free mice showed that the lack of regulatory effects of microbiomes leads to abnormal brain development in mice, such as abnormal growth of microglia, high myelinization of the prefrontal cortex, and increased permeability of the blood-brain barrier (Clarke et al., 2013; Braniste et al., 2014; Thion et al., 2018). In microbial deficiency mice, the expression of genes related to neural development is affected, including neuronal plasticity and neurotransmission in the hippocampus (Stilling et al., 2015; Chen et al., 2017). These changes in neurophysiology will eventually translate into anxiety, increased stress response, cognitive deficits, visceral pain response, and changes in fear (Gareau et al., 2011; Luczynski et al., 2017; Hoban et al., 2018). In addition, studies showed that changes in the structure of perinatal microbiomes regulate gene expression, function, and morphology of progeny microglia, these effects will appear in the early stages of embryo development. These data reveal that the colonization of microbiomes in early life has a huge impact on the neural development and function of offspring.

In terms of genetic research about maternal microbiomes, experiments indicated that microglial status can be regulated in microbially depleted mice during adulthood, and three independent studies emphasized the role of maternal microbiomes in guiding embryonic microglial development. What's more, the gene expression changes of germ-free mouse microglia were more obvious in adult microglia than in neonatal microglia compared with the conventional control group (Matcovitch-Natan et al., 2016). These findings suggest that microbiomes are important for microglia development from adulthood to adult phenotype. Nonetheless, 240 genes are expressed differently even in newborn microglia, suggesting that the maternal microbiomes may direct microglia maturation during prenatal development. Consistent with this, 19 differentially expressed genes were detected in microglial cells harvested from germ-free embryo (14.5-day-old), indicating that the maternal microbiomes have little effect on microglial progenitor cells (Thion et al., 2018). So, why are there differences of opinion on the effects of maternal microbiomes on offspring? The main reason is that the research phase of pregnancy is different. It is now widely believed that the moment of birth is the first opportunity for large-scale colonization of neonatal bacteria (Biasucci et al., 2008; Backhed et al., 2015). Therefore, the mode of delivery has a huge impact on the establishment of infant microflora. Numerous studies have begun to link childbirth patterns to the unique trajectories of neonatal microbial development (Biasucci et al., 2008; Backhed et al., 2015). Studies revealed that newborns born by cesarean section (without exposure to the birth canal) at birth are unable to obtain vertical transmission of bacteria and viruses from the mother's vagina (Backhed et al., 2015; McCann et al., 2018). In addition, it should be emphasized that the changes in the progeny microbial structure due to different delivery methods are temporary. However, the composition and abundance of intestinal micro-organisms in infants born by vaginal delivery are significantly higher than those in newborns born by cesarean section (Biasucci et al., 2008; Azad et al., 2013; Jakobsson et al., 2014; McCann et al., 2018).

So, how do microbiomes affect the neurodevelopment of offspring? The possible mechanisms are as follows: (1) Toll-like Receptors (TLRs) signaling on neuronal proliferation: TLRs can perform innate immune recognition on the components of microbiomes; TLRs are expressed in all subtypes of brain resident cells, including intact expression in astrocytes, neurons, and oligodendrocytes (Hanke and Kielian, 2011; Kawai and Akira, 2011). (2) Cytokine signaling on neurogenesis: In the brain, cytokines have different effects on neurodevelopment. For example, IL-4 inhibits the proliferation of mouse embryonic neural precursor cells (NPCs), IL-34 and CSF-1 promote neuronal proliferation, and IL-6 promotes the occurrence of fetal striatum cells; maternal intestinal microbiomes can fully change germ-free mouse intestinal cytokines distributed (Pronovost and Hsiao, 2019). (3) Complement proteins in synaptic refinement: The complement system contributes to the clarity of cells and humoral-mediated pathogenic microbiomes (Ricklin et al., 2016). Synaptic complement proteins play an important role in the early development of neurons. For example, complement protein C3 is localized to the axons of retinal geniculate cells and depends on upstream complement proteins C1 and C4 (Sekar et al., 2016).

## Conclusion

In summary, GMs play a vital role in a variety of complications in pregnant women. Current researches reveal that the abundance and composition of some GMs are altered during pregnancy complications. Maternal GMs could affect pregnant women's physiological metabolism, immune system or inflammatory response (**Table 2**). However, in terms of impact on offspring, it is thought that the effect of maternal GMs on the fetus is not significant. Maternal microbiomes that affect offspring are mainly those in the vagina or milk. In addition, the mode of childbirth is also important for the colonization of microbiomes in the baby, because the moment of birth is the first opportunity for large-scale colonization of neonatal bacteria. Nevertheless, the transmission of maternal microbiomes is closely related to the offspring's immune system and neurodevelopment. Therefore, exploring the role and mechanism of GMs in pregnancy complications and offspring is very meaningful for managing their health. The limitation here is that most of the current studies on the role of maternal microbiomes in pregnancy complications or offspring are only relevance studies, and still in infancy, further mechanistic and interventional trials or prospective studies are needed.

**Table 2:** Changes of gut microbiomes in pregnancy complications and its mechanisms.

Complications of pregnancy	Increased abundance of microorganisms	Decreased abundance of microorganisms	Mechanisms	References
Gestational diabetes	*Firmicutes, Klebsiella variicola, Collinsella, Rothia, Ruminococcus, Actinobacteria, Parabacteroides distasonis, Desulfovibrio*	*Parabacteroides, Dialister, Akkermansia, Roseburia, Methanobrevibacter smithii, Eubacterium species, Alistipes species, Bacteroides Bifidobacterium species*	Intestinal permeability, anti-inflammatory cytokine levels, etc.	Bassols et al., 2016; Navab-Moghadam et al., 2017; Crusell et al., 2018; Ferrocino et al., 2018
Gestational obesity	*Bacteroides, Bifidobacterium*	*Enterobacteriaceae, Escherichia coli*	Microbial metabolites, inflammatory responses, epigenetic modifications, etc.	Santacruz et al., 2010; Barlow et al., 2015; Khan et al., 2016; Soderborg et al., 2016; Gu et al., 2017; Collado et al., 2008
Preeclampsia	*Bulleidia moorei, Clostridium perfringens*	*Coprococcus catus*	Future research is needed	Lv et al., 2019
Disease of digestive tract	*Acinetobacter*, *Enterococci*, *Paenibacillus*	*Blautia, Collinsella*	Future research is needed	Jin et al., 2019
Autoimmune disease	*Firmicutes, Acholeplasmatales, Desulfovibrionales*	*Verrucomicrobia, Bacteroidales*,	Autoimmune response	Pan et al., 2019

## Author Contributions

YY and XC drafted the manuscript. CC, YZ, HF, and FC modified the manuscript. WF and CZ edited and added valuable insights into the manuscript. All authors approved the final manuscript and agreed to be accountable for all aspects of the work.

## Funding

This study was supported by the National Natural Science Foundation of China (Grant Nos. 81873838, 81802630).

## Conflict of Interest

The authors declare that the research was conducted in the absence of any commercial or financial relationships that could be construed as a potential conflict of interest.
